# The Role of Interproximal Reduction (IPR) in Clear Aligner Therapy: A Critical Analysis of Indications, Techniques, and Outcomes

**DOI:** 10.7759/cureus.56644

**Published:** 2024-03-21

**Authors:** Feras Y Dahhas, Nawaf S Almutairi, Rayan S Almutairi, Husam A Alshamrani, Hammad S Alshyai, Rayan K Almazyad, Munerah S Alsanouni, Safa A Gadi

**Affiliations:** 1 Dentistry, Al-Noor Specialist Hospital, Makkah, SAU; 2 Dentistry, Qassim University, Ar Rass, SAU; 3 Dentistry, King Fahad Military Medical Complex, Dhahran, SAU; 4 Pediatric Dentistry, Ministry of Health, Riyadh, SAU

**Keywords:** orthodontic therapy, interdental enamel reduction, interdental papilla, clear aligner, orthodontics treatment

## Abstract

Interproximal reduction (IPR) has become a standard practice in orthodontic treatment, particularly in the clear aligner therapy. It became an integral part of the digital plan when using clear aligners. Given the irreversible nature of IPR, precise planning and performance is essential. This article aims to analyze and summarize the existing literature on IPR in the context of clear aligners. The goal is to help clinicians to gain essential knowledge for safely and effectively navigating IPR. The review critically examines different perspectives found in the literature, covering indications, methods, and outcomes. Topics exploring the impact of IPR on treatment outcomes include space gaining, addressing tooth size discrepancies, tooth shape adjustments, resolving malocclusion, and enhancing aesthetics. Emphasizing precision of the procedure by the clinician and awareness of contraindications, the article also discusses the impact of IPR on patients. This includes considerations like increased pulp temperature, susceptibility to cavities due to changes in enamel roughness, effects on soft tissues, and post-IPR tooth sensitivity.

## Introduction and background

Interproximal reduction (IPR), the reduction of mesiodistal dimensions of the teeth, is now an almost routine part of orthodontic treatment, especially in adult patients [1]. The procedure was first formally described by Sheridan in 1985 [2] and can be found under other terms like interdental or interproximal stripping, slenderizing, reduction, selective enamel reduction, interdental enamel reduction, or enamel approximation. In the context of clear aligner therapy, the integration and extent of IPR for each tooth are typically determined during the digital planning phase [3], made possible by technological advances in this area [4-5]. Since IPR is an irreversible change made to the tooth tissue that does not have the ability to regenerate, it is, therefore, crucial to plan and perform the procedure correctly. A comprehensive understanding of the prescribed protocols, appropriate indications, and inherent constraints is essential for attaining the intended outcomes. Various aspects, spanning from methodology to indications and outcomes, have undergone thorough scrutiny in the scientific literature, yielding sometimes diverse conclusions. Thus, the objective of this review is to conduct a critical analysis of the role of IPR in clear aligner therapy, recognizing that due to varying perspectives, determining the most accurate approach can be challenging for practitioners. This article aims to provide readers with the necessary knowledge to navigate IPR safely and effectively while addressing the controversies and considerations associated with IPR in clear aligner therapy.

## Review

Indications

The primary indication for interproximal reduction (IPR) discussed is the need to create space within the dental arch [1]. Subsequently, other topics addressed include resolving tooth-size discrepancies, tooth reshaping, correcting malocclusions, enhancing aesthetics, and improving stability.

Space-gaining procedures

Enamel reduction serves as an alternative approach to create the necessary space for aligning misaligned teeth. It is especially beneficial for addressing anterior segment crowding ("social six"), most often incisors. Minor to moderate crowding (4-8 mm) is the main reason for IPR in the orthodontic treatment of adults [1,6]. However, Fiori et al. consider IPR to be the least predictable among the treatment options for space gain with clear aligners when compared to the sagittal inclination of incisors and changes in arch diameter, with the predictability of 49% for the upper arch and 42% for the lower arch [7].

As the non-extraction approach in orthodontic treatment has become highly favored compared to extraction-based protocols, replacing the use of extractions by using IPR results in shorter overall tooth movement distances and diminishes the risk of residual gaps that may occur in extraction cases [8-9]. Utilizing IPR in conjunction with distalization results in a reduced need for distalization to achieve a Class I occlusion, consequently decreasing the number of aligners necessary for treatment [10]. The precise quantification of approximal enamel suitable for removal through IPR proves challenging due to the inherent variability in enamel width among both individuals and studies [11]. Moreover, the range of enamel thickness deemed as "safe" for removal has been observed to expand progressively over time [12]. It appears that the enamel is about 0.1 mm thicker on the distal side of the tooth [11], and there is no proven correlation between enamel thickness and tooth size [6]. The presence of 1 mm of enamel per approximal side in the frontal section is not consistently encountered; however, it is observed from the surface of the canine onward [6,11].

Although the consensus acknowledges that 50% enamel coverage is deemed adequate for tooth protection [13], Zachrisson et al. claim that more significant grinding does not have a harmful effect on the tooth [14-15]. Employing the IPR procedure in lateral segments is expected to result in 8 mm of space per arch [8,16] or even more if we go to the maximum possible values in the lateral segment [17]. In the frontal area, we should not exceed removing 0.5 mm from the lower incisors and upper lateral incisors [16]. Frindel suggests better not to remove more than 0.3 mm of the enamel from the upper incisors and 0.2 mm from the lower incisors [6].

Tooth size discrepancies

The upper-to-lower tooth size ratios and indexes proposed by Bolton and published in 1958 [18] are now widely known and still have an important place in orthodontics. The anterior ratio reaches values of 77.2 ± 1.65%, and the posterior ratio reaches 91.3 ± 1.9% [18]. Research findings indicate that the number of individuals with notable anterior tooth-size discrepancies ranges from 20% to 30%, while the prevalence of overall tooth-size discrepancies falls between 5% and 14% [19]. Noar and Kneafsey declare that IPR allows to normalize Bolton‘s index to produce the correct overbite and overjet at the end of treatment (an alternative would be to leave a gap distal to the lateral incisor or on either side of it) [20]. Sometimes, following the extraction of the lower incisor, it may be necessary to perform a slight interproximal reduction on maxillary incisors to ensure the maintenance of adequate overbite and overjet [10]. Conversely, if IPR needs to be performed in a patient with correct tooth proportions, Heusdens et al. indicate that even in cases where patients exhibit an abnormal Bolton ratio, the teeth can generally be repositioned to achieve a satisfactory occlusion [21].

Tooth reshaping

Tooth reshaping is most often performed in the visible part of the dentition, that is, on the incisors or canines. The reason for the need to change the shape of the tooth is aesthetics or improvement of the shape [14-15]. Certain rules must always be followed when changing the approximal shape of a tooth. Tarnow et al. recommended maintaining an interproximal contact distance of 4.5 to 5 mm from the upper margin of the alveolar crest to prevent the visibility of 'black triangles' in the frontal area [22], which in healthy periodontium is about 1.5-2 mm apical to cementoenamel junction. Therefore, it is crucial to focus on shaping the contact points correctly for teeth undergoing planned IPR [23]. During tooth recontouring through IPR, it is imperative to ensure optimal access to the contact points and to maintain a clearly visible long axis of the tooth.

In the upper incisal area, the most common shapes can be categorized into three types: triangular, barrel shaped, and rectangular [24]. Each type is different and suitable for reshaping. Teeth with a triangular morphology are considered the most suitable candidates for IPR, as even a small reduction of enamel creates significant space. Barrel-shaped teeth typically feature contact points located toward the middle of the tooth, leaving apparent space at the incisal edges. While enamel reduction may help approximate the incisal edges, it may also result in the relocation of the contact point apically. Rectangular shapes pose a higher risk of creating ledges and suboptimal contact points [1,23].

Frequently, we encounter the need for reshaping of the upper canines. There are cases where the canine is placed at the position of the lateral incisor [25]. If the reshaping is performed correctly, even a significant change in shape requiring the removal of a large amount of enamel does not lead to tooth damage, pulp obliteration, or other changes and leads to a long-term stable and predictable result [14-15,25]. Regarding lower incisors, tooth shape, specifically the mesiodistal (MD) and faciolingual dimensions (FL), may play a role in the potential occurrence of lower incisor crowding [26]. Peck and Peck capitalized on this insight to devise an index applicable to clinical orthodontics [26]. This index employs an MD/FL ratio to assess whether a lower incisor possesses a favorable or unfavorable shape for achieving optimal lower anterior alignment. The recommended MD/FL index values for lower incisors fall within the range of 88% to 92% for the mandibular central incisor and 90% to 95% for the mandibular lateral incisor. Enamel reduction can, therefore, fine-tune the index values so that they are within the specified ranges [26].

Resolving malocclusion

In some cases, properly directed IPR can facilitate tooth movement toward the desired occlusion: IPR can be used in Class I arch-length discrepancies, for example, in cases where molars maintain a Class I relationship, while canines exhibit a mild Class II relationship. The implementation of IPR in the molar and premolar regions facilitates the distal movement of canines, thereby optimizing the cusp-to-fossa relationship [10].

**Figure 1:**
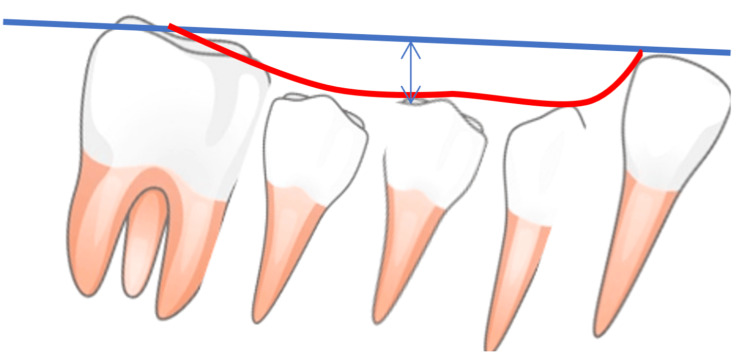
Showing deep curve of Spee which can be flattened leading to an increase in arch length The curve of Spee's depth is measured by drawing a line from the lower canine to the second molar's distobuccal cusp and then measuring the vertical distance to the curve's lowest point, in millimeters. Image credits: Feras Y. Dahhas.

IPR is particularly well-suited for addressing minor anteroposterior corrections, both Class II and Class III. In the case of Class II, the principle is to retract the maxillary canines to Class I and reduce the amount of distalization of the upper molars required. We can facilitate such movements by posterior IPR in the upper arch [1]. Vice versa, in cases of Class III, the goal of IPR in the lateral sections is to retract the lower incisors and reduce the amount of distalization of the lower molars needed [10]. If midline correction is necessary, IPR offers a predictable outcome. It is recommended to check midlines always on real patients (in vivo), especially in cases where dental midlines align but deviate from the facial midline [10]. Correction of the curve of Spee is also possible [1]. Furthermore, evaluating the efficacy of IPR in the management of severe malocclusion treatment or complicated cases is challenging due to the frequent absence of comprehensive data on the extent of IPR conducted in studies [27] (Figure 1).

Aesthetics of gingival contour

**Figure 2:**
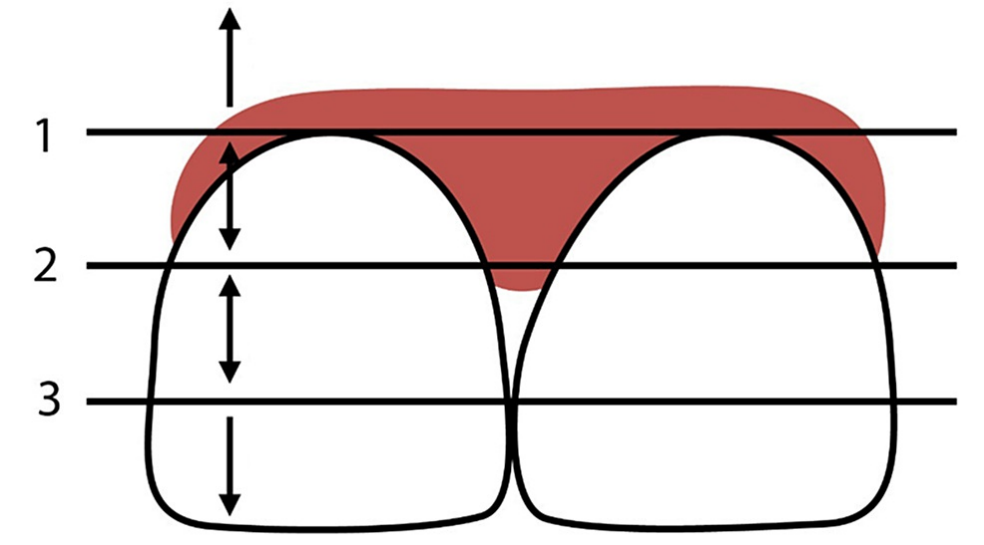
Categorization of open gingival embrasures 1: Line passing through the most cervical contact point. 2: Line bisecting the distance between lines 1 and 3. 3: Tangent to the apical-most aspect of the clinical crown. Below line 1: No papilla shown. Between lines 1 and 2: Moderate loss of papilla. Between lines 2 and 3: Mild loss of papilla. At line 3: Optimal level. Image credits: Feras Y. Dahhas.

In 1997, Jemt defined a papillary index, establishing the optimal condition, wherein the papillae completely occupy the interdental space and extend to the gingival aspect of the tooth contact site [28] (Figure 2).

The deficiency of papillae within the interdental space, characterized by the manifestation of black triangles, is regarded as aesthetically undesirable. This phenomenon may be pre-existing in the patient prior to treatment initiation, or black triangles may emerge during the treatment, particularly following the alignment of rotated incisors [29]. In the study conducted by Zhang et al., upon comparing the occurrence of black triangles between fixed appliance and clear aligner therapies in non-extraction cases, a heightened incidence of gingival embrasure opening was observed undergoing clear aligner therapy, with rates of 25.7% for maxillary incisors and 40.3% for mandibular incisors [30]. In contrast, fixed appliances exhibited incidences ranging from 22% to 36% [31]. One prospective method for reconstructing the diminished interdental papilla involves apically shifting the contact point of the teeth by changing the contour of the tooth crown. If the shape of the tooth is suitable, IPR has the capacity to prevent or minimize retraction of the interdental gingival papilla [29].

Stability

Addressing stability concerns as a critical consideration in orthodontic treatment. It is noteworthy that flaring the mandibular incisors beyond a certain limit has proven to be an unsuccessful treatment strategy [32]. Germec-Cakan states that IPR in the frontal area helps to preserve intercanine arch widths and also the arch perimeter in Class I borderline patients with moderate crowding [33]. Alpakan et al. reported comparable stability in incisor alignment when IPR was employed compared to cases without IPR [34]. This finding contrasts with the outcomes of the Peck and Peck study discussed earlier in the section on Tooth Reshaping, addressing the MD/FL ratio [26].

Aasen and Espeland explored the potential of employing IPR to stabilize the contact points, avoiding the need for a fixed retainer [35]. Their protocol involved early-stage over-correction of rotated mandibular incisors and stripping during treatment and follow-up. The outcomes demonstrated favorable stability three years post-treatment [35]. However, further research is needed to clarify the effect of IPR on the stabilization of contact points and tooth position [36].

Contraindications

Contraindications for IPR documented in the literature are the following. IPR is not advisable in situations where the required reduction surpasses the recommended limit for a specific arch or tooth type [8]. Consideration should also be given to prior instances of IPR [13]. Additionally, it is important to exercise caution when considering IPR for individuals with poor oral hygiene, active periodontal disease, teeth hypersensitivity, enamel hypoplasia, or in cases where the teeth are naturally small [1,6,8,15]. Tooth with a rectangular or square shape may not be ideal candidates due to the potential risk of creating ledges. Such tooth shapes often necessitate extensive IPR to establish adequate space and generate broad contact surfaces, which may result in food impaction and diminished interseptal bone [1,8]. Contraindications are also associated with large pulp chambers, particularly in younger patients [1,8]. We do not perform the IPR where teeth are rotated to the extent that they prevent proper access to the contact area, even with the use of separators [15,23], including significant crowding [1,8].

Techniques

This section examines IPR techniques in orthodontics, highlighting both well-described methods and subtle variations that contribute to the diversity in practice.

General recommendations

According to literature recommendations, we consistently adhere to the principle of planning the removal of enamel in the most conservative manner, ensuring that only the minimal amount necessary is designated for removal. This includes the need for anchorage planning so that the space gained is not lost by unwanted tooth movement [16]. Prior to commencing treatment, it is prudent to obtain signed informed consent from the patient or legal guardian [8]. IPR is only performed in patients with good dental hygiene and a low risk of dental caries [1,6].

**Figure 3:**
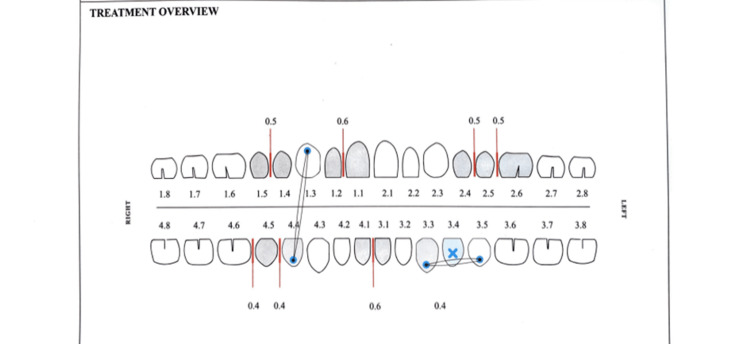
A sample form for interproximal reduction Image credits: online form.

When using clear aligners, the clinician receives the prescribed amount of IPR, usually 0.2 to 0.5 mm per contact area [10]. It is also possible to remove the desired amount of enamel gradually over several visits [16,10], and it is advisable to perform IPR when the teeth are already aligned, despite the possibility of minor round-tripping [10,37]. It is recommended to refrain from performing IPR prior to bonding attachments to the teeth [6] (Figure 3).

Instruments

Accepted instruments include using hand instruments and mechanical handpieces. We can use abrasive metal strips handheld or stretched in a manual holder. To speed up chair time, we can use air-rotor stripping (ARS) with files (oscillating), diamond discs, or burs (rotatory). Diamond discs are single or double-sided, mounted on a straight slow-speed handpiece, and are meant for interior use only. It is possible to use a clear adaptor to shield the surrounding tissues. Fine burs are mounted in a high-speed handpiece [17], and it is beneficial to use safe-tipped ones [16]. When comparing manual strips, discs, and bur for enamel stripping, Nassif et al. found an abrasive metal strip most effective in replicating the shape of the proximal tooth surface [38].

Variously thick paddles, called IPR gauges, are used to measure the space gained during IPR [8,16]. To improve the visual clarity of the working field, elastomeric separators can be used, which are inserted into the interdental space in the frontal section for two to four days and in the lateral section for one week [8]. Although not usually necessary, topical anesthesia can be used in sensitive individuals [8].

It is advisable to choose a manual instrument for smaller increments; on the other hand, if a large IPR is required, it is better to choose a disc or bur [10]. Chudasama and Sheridan recommend the preservation of soft tissue by inserting a 0.020-0.030" brass or steel wire into the interdental area while performing the ARS [16]. When using separators, it is important to measure the space created by the separator after removing it (before starting the IPR) to add the dimension to the target value. If separators are not used, breaking the contact points should be done manually before switching to mechanical handpieces [8].

Finishing and polishing

After removing the desired amount of enamel and obtaining the necessary space, the enamel needs to be finished and polished by finishing strip and polishing file [8-16]. There is considerable variation in the reported levels of enamel roughness after IPR with or without polishing [39]. Even after polishing, scratches may be visible on the teeth [40-41]. This is the reason Joseph et al. recommend smoothing the surfaces using phosphoric acid gel [40], which should then be rinsed off with a water spray [16].

Subsequently, application of fluoride gel is recommended to encourage remineralization [8-16]. Alessandri Bonetti et al. proved in vitro that the utilization of a toothpaste containing zinc-carbonate hydroxyapatite appears to be another effective method for safeguarding stripped enamel surfaces from demineralization [42]. Resin infiltration represents another potential care strategy following IPR [43]. Almansouri et al. concluded that the use of minimally invasive (MI) varnish, a casein phosphopeptide-amorphous calcium phosphate (CPP-ACP) containing varnish, proved to be the most effective agent in protecting proximal enamel surfaces after IPR against acidic attack. The MI varnish demonstrated significant improvements in enamel resistance compared to the other groups, including Icon resin infiltration, and fluoride-treated groups [44].

Outcomes

This section delves into the comprehensive analysis of outcomes associated with IPR in orthodontic treatment, exploiting findings from the existing literature.

Increase in dental pulp temperature

Utilizing mechanical or manual diamond instruments in IPR has been shown to elevate the temperature of the dental pulp, with diamond burs and discs exhibiting the most pronounced effect, particularly when operated at the maximum recommended speed without coolant. This observed temperature elevation can achieve statistical significance. However, the study conducted by Omer and Al Sanea demonstrated that all recorded temperatures remained below the critical threshold of 5.5°C [45]. The same conclusions were confirmed in vivo in the study by Banga et al. [46]. This finding underscores the importance of conscientious tool selection and operational parameters to mitigate potential thermal effects on dental pulp [45]. It is imperative to emphasize that during IPR, particularly when employing mechanical instrumentation, a discontinuous approach is essential. For instance, prolonged contact of a bur with a tooth, even for as little as 10 seconds, can generate a temperature that poses a risk of causing irreversible harm to the dental pulp [47].

Changes to enamel surface and susceptibility to tooth decay

The conclusion of the 2014 systematic review was that the outcomes following the use of various methods of IPR are hard to interpret but overall reveal that the treated surface tends to exhibit increased roughness [39]. Although Butrus and Chawshli [48] did not identify a significant distinction in enamel nano topography between polished and non-polished surfaces, the polished surface did exhibit somehow smoother texture. Therefore, post-IPR polishing is recommended [49-50].

Susceptibility to tooth decay has been disproved, as confirmed by a systematic review from 2014 [39] and a newer review from 2022 [9]. This refers to both the mere demineralization of enamel and the occurrence of caries itself [9]. While adolescents may occasionally encounter challenges in sustaining dental hygiene, particularly in interdental care, it is generally observed that patients, and specifically adults undergoing treatment, exhibit a propensity for maintaining exemplary oral hygiene [15]. Nonetheless, if the procedure is not executed accurately, there is a possibility of inadvertently causing damage to an adjacent tooth. In this context, the potential iatrogenic consequences include an elevated risk of caries, periodontal issues, and heightened temperature sensitivity in these damaged teeth [17].

Periodontal and bone health

In the evaluation of cone beam computed tomography (CBCT) scans before and after treatment, Hellak et al. in their study observed no significant impact of IPR on interradicular space condition [51]. Furthermore, their findings indicate an increase in root distance post-treatment, resulting in augmented bone volume; however, this effect is not attributable to IPR [51]. The absence of periodontal problems is also confirmed by a systematic review from Gómez-Aguirre et al. [9]. Shalchi et al. in their retrospective study likewise excluded an effect of IPR on attachment loss and bleeding on probing [52].

Hypersensitivity

Tooth sensitivity exhibits a degree of subjectivity and depends on factors such as the age of the patient, the extent of crowding, pre-existing sensitivity, and the amount of enamel removed during the procedure [1]. In their 2017 publication, Meredith et al. assert that the existing literature does not indicate any enduring impact on dental sensitivity subsequent to IPR [12]. A congruent conclusion is reached by Gómez-Aguirre et al. in their 2022 systematic review, affirming that IPR does not escalate tooth sensitivity [9].

Precision in IPR in clear aligner therapy

An interesting topic is the clinical experience with how much stripping is actually performed on patients. The actual amount of enamel removed during procedures conducted on the patient can deviate from the initially intended interproximal reduction (IPR) in virtual planning. Research shows that in most cases, the executed IPR is lower than what had been initially anticipated [53-54]. Hariharan et al. found that the amount of implemented IPR is consistently lower than the digitally programmed values, particularly noted in mandibular anterior teeth and maxillary posterior teeth [55].

## Conclusions

IPR has an undeniable role in the treatment with clear aligners. It is used when space gain is needed to address tooth size discrepancies, to adjust tooth shape as part of the resolution to malocclusion, to improve the aesthetics of the gingival contour, and to improve stability. All these topics are widely discussed in the literature. It is crucial to perform IPR with the correct procedure and avoid contraindications of the treatment. Furthermore, it is advisable to be aware of the effects of IPR on the patient; issues discussed include the increase in pulp temperature, susceptibility of the tooth to caries related to the change in the roughness of the stripped enamel surface, soft tissue health, and hypersensitivity of the teeth after IPR. All of these are related to the precision of the clinician during the procedure.
